# Validity of self reported male balding patterns in epidemiological studies

**DOI:** 10.1186/1471-2458-4-60

**Published:** 2004-12-13

**Authors:** Rosalind Taylor, Julia Matassa, Justine E Leavy, Lin Fritschi

**Affiliations:** 1 School of Population Health, University of Western Australia, Perth, Australia

## Abstract

**Background:**

Several studies have investigated the association between male pattern baldness and disease such as prostate cancer and cardiovascular disease. Limitations in the lack of standardized instruments to measure male pattern baldness have resulted in researchers measuring balding patterns in a variety of ways. This paper examines the accuracy and reliability of assessment of balding patterns by both trained observers and men themselves, using the Hamilton-Norwood classification system.

**Methods:**

An observational study was carried out in Western Australia with 105 male volunteers aged between 30 and 70 years. Participants completed a short questionnaire and selected a picture that best represented their balding pattern. Two trained data collectors also independently assessed the participant's balding pattern using the same system and the men's self assessment was compared with the trained observer's assessment. In a substudy, observers assessed the balding pattern in a photo of the man aged 35 years while the man independently rated his balding at that age.

**Results:**

Observers were very reliable in their assessment of balding pattern (85% exact agreement, κ = 0.83). Compared to trained observers, men were moderately accurate in their self-assessment of their balding status (48–55% exact agreement, κ = 0.39–0.46). For the substudy the exact agreement between the men and the observers was 67% and the agreement within balding groups was 87%.

**Conclusions:**

We recommend that male balding patterns be assessed by trained personnel using the Hamilton-Norwood classification system. Where the use of trained personnel is not feasible, men's self assessment both currently and retrospectively has been shown to be adequate.

## Background

Male pattern baldness is the most common form of baldness observed in human beings[1]. The two main types of balding are: frontal balding in which the hair recedes bilaterally from the forehead region backwards; and vertex balding in which a bald spot appears on the top back of the head. Total balding may be due to continued spread of frontal balding, or a joining up of frontal recession and vertex balding[1,2]. Whilst human hair growth is affected by a number of factors, androgens are the most obvious regulators of normal hair growth and are a prerequisite for male pattern baldness[1]. Serum levels of total and free testosterone, sex hormone binding globulin, and dihydrotestosterone may be important especially given the strong association between free testosterone level and baldness[3].

Several studies have been conducted investigating the association between male pattern baldness (as a proxy for testosterone levels) and various health issues such as prostate cancer [3-7] and cardiovascular disease[6,8,9]. Different methods have been used to assess male pattern baldness in these studies, including the Hamilton-Norwood classification system[2]. This study was undertaken in order to determine the accuracy and reliability of assessment of balding patterns by both trained observers and men themselves, using the Hamilton-Norwood classification system[2].

## Methods

Subjects were male volunteers between the ages of 30–70 years recruited from sporting clubs, shopping centres, universities, the central business district and other public areas throughout the metropolitan area of Perth, the capital city of the state of Western Australia, during March 2003 to August 2003. Of the 105 volunteers recruited, two were less than 30 years of age, and therefore were ineligible for the study.

**Figure 1:**
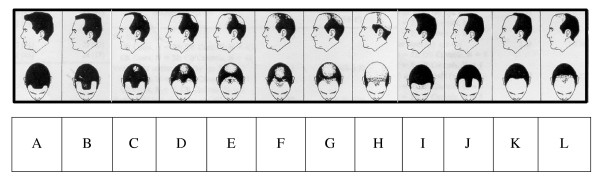
Hamilton Norwood Classification Scale (OT Norwood, 1975)

Participants completed a questionnaire providing minimal information regarding demographic characteristics, including age, ethnicity, and level of education. Included in the questionnaire was an unlabelled Hamilton-Norwood classification scale[2] (Figure 1), which participants were asked to examine and to select the picture they believed best depicted their own balding pattern.

Whilst the subjects completed the questionnaire, two data collectors with some training in recognising different balding patterns independently assessed the participant's balding pattern using the same classification system. On recruitment, participants were told the study was to help with medical research, however the role of the data collectors with regard to their independent assessment of the participant's balding pattern was not revealed until after the questionnaire was complete.

Where possible, (sporting clubs and universities) volunteers were asked to bring a photograph of themselves at age 35 on the day on which the study would be done. The photographs were used by the observers to independently assess the men's balding patterns at age 35 while the participants completed the questionnaire. These participants were also asked to select the picture from the Hamilton-Norwood scale which best depicted their balding pattern at age 35, and were not permitted to look at the photographs while completing the questionnaire.

For data analysis, we compared the assessments of the two observers with each other, and the assessment of the participants with the assessments of the observers. Initially, each of the 12 reference pictures included in the Hamilton-Norwood classification system[2] were considered separately and the percentage of cases with exact agreement on these was determined between each of the observers and the subjects, and between the two observers. The classifications were then arranged into four groups according to overall balding pattern: no balding (A and B in Figure 1); vertex balding only (C); combination of frontal and vertex balding (D to H); and frontal balding only (I to L). Percent agreement within group was then calculated for each observer versus the subjects and between the two observers. The percent of agreement was compared by: age groups (younger than 50 years, and 50 years or older); self-reported ethnicity ("Australian" versus other); and education. Where possible, kappa statistics were also calculated.

## Results

There were 69 participants (67%) between the ages 30–49 years, and 34 (33%) were between the ages 50–70 years. Whilst 70% of subjects considered themselves of "Australian" ethnicity (Caucasian born in Australia), the remainder was made up of representatives from Europe (20.4%), Asia (4.9%), New Zealand (3.9%) and South America (1%). A third of subjects had trade education or equivalent (30.1%); 22.3% had completed high school but not gone onto further education; 12.6% had completed junior high school only; and, because of recruitment through universities, 19.4% of subjects had an undergraduate degree and a further 15.5% had completed post-graduate study.

Overall, trained observers were found to be highly reliable at analysing balding patterns with an exact agreement of 85.4% and an agreement within balding pattern groups of 90.3% (Table 1). Compared to the observers, men were found to be moderately accurate in their ability to describe their current balding pattern with an exact agreement percentage ranging from 48.5 to 55.3% and agreement as to balding group around 70%.

**Table 1:** Reliability and validity of assessment of balding patterns

	**Exact**		**Balding groups**	
	**% agreement**	**κ **(p)	**% agreement**	**κ **(p)

Observer 1 vs. Observer 2	85.4	0.828 (p < 0.001)	90.3	0.858 (p < 0.001)
Observer 1 vs. subjects	48.5	0.386 (p < 0.001)	68.0	0.520 (p < 0.001)
Observer 2 vs. subjects	55.8	0.463 (p < 0.001)	73.8	0.599 (p < 0.001)

In regards to how different demographic characteristics affect men's ability to predict their balding pattern (Table 2), the characteristic with the most influence appeared to be age, with men aged 50 or above being more accurate (exact agreement 56–62%) than men aged less than 50 years (exact agreement 45–52%). Men who finished high school were the most accurate at assessing their balding status followed by either those that had studied at technical college or university. The least accurate were men who had completed year 10 at high school or less, with men who had completed post-graduate studies also performing fairly poorly. The effect of ethnicity on ability to assess balding patterns (between Australian and non-Australian men) appeared to be of little significance.

**Table 2:** Validity of assessment of balding group by age, ethnicity and education.

	**Observer 1 vs subjects**		**Observer 2 versus subjects**	
	**% agreement**	**κ **(p)	**% agreement**	**κ **(p)

Age group				
<50 years	65.2	0.46(<0.001)	72.5	0.56(p < 0.001)
>50 years	73.5	0.62(<0.001)	76.5	0.66(p < 0.001)
Education				
Junior high school	46.2	0.29 (p = 0.058)	61.5	0.48 (p = 0.003)
Senior high school	91.3	0.86 (p < 0.001)	87.0	0.79 (p < 0.001)
Trade school	64.5	0.46 (p < 0.001)	74.2	0.58 (p < 0.001)
Undergraduate	70.0	-	70.0	-
Postgraduate	56.2	0.37 (p = 0.013)	68.7	0.57 (p < 0.001)
Ethnicity				
"Australian"	66.7	0.50 (p < 0.001)	75.0	0.61 (p < 0.001)
Other	71.0	0.57 (p < 0.001)	71.0	0.57 (P < 0.001)

There were 15 subjects who provided photos of themselves aged approximately 35 years and both observers examined 13 of these. The inter-observer reliability for exact match was 81.8% (κ = 0.766, p < 0.001) and agreement within balding pattern groups increased to 100% (κ = 1, p < 0.001). Observer 2 only examined 13 subject, so for the 15 subjects examined by Observer 1, agreement between the men and the observer was 66.7% for exact match and 86.7% for agreement within the balding pattern groups.

## Discussion

In this study we have shown that trained observers are very reliable in assessing men's balding patterns. Our data also show that, when compared to the trained observers, men themselves can assess their balding patterns quite well. In particular, men are accurate in assessing which balding pattern group they have. This result is important due to previous research suggesting that it is the overall pattern of hair loss rather than extent of balding that determines whether men are at an increased risk of developing negative health outcomes including prostate cancer[3,4,7].

There have been several studies investigating the link between male pattern baldness and prostate cancer[3-5,7,10], as well as other health issues such as cardiovascular disease[6,8,9]. In these studies, balding patterns have been assessed using different techniques, some more complex than others. There has been controversy over the use of some of the more simplistic methods of assessment[6], as little research has been performed regarding their accuracy and their ability to discriminate between the types of balding (vertex, frontal, and combination of vertex and frontal). In a study performed by Hererra *et al*[11] assessment of subject's balding pattern involved counting the total number of bald spots found on the head. In a repeat assessment performed six years later on the same subjects, there was actually a decreased level of baldness in 12% of study participants. This apparent reversal of baldness was unable to be attributed to regrowth from treatment or other means, and so it must be concluded that the methods used to assess baldness in these participants were unreliable.

Other methods for assessing baldness have been used in clinical situations including reference grids used with standardized photographs of the scalp or in vivo[12]; and videomicroscopic[13] and macrophotographic[14] techniques in which the individual hairs are counted. While these techniques may be used in well-funded clinical trials with the aim of assessing change in balding, they are not appropriate for epidemiological studies in which often the only requirement is to classify men as to their type of balding.

The majority of studies[4,7,10,15] have used variations on the Hamilton scale as modified by Norwood in 1975[2]. This method allows for the grading of baldness in terms of severity and pattern. The scale can be used either by independent observers, or by men themselves, but no official instructions for use or training manuals are available. No previous studies have been performed to assess the accuracy and reliability of either trained independent observers or the participants themselves in the assessment of balding patterns.

The strengths of our study included the recruitment of volunteers from a broad cross-section of the population thus allowing for extrapolation of the results back to the wider community. As our research was performed as an observational study, care was taken to avoid the data collectors influencing the results. This included not informing participants that the observers would be assessing their balding pattern until after the questionnaire was complete. The data collectors also refrained from giving advice to participants when asked to help assess their balding pattern.

Older subjects appeared to be better at assessing their balding group than men less than 50 years. This may be due to greater hair loss resulting in a more straightforward distinction between balding patterns, or possibly just a greater self-awareness of degree of balding amongst those in the older age group. The results with regard to education were confusing with men who had only senior high school education seeming to be best at assessing their balding patterns. This may have just been due to small numbers in the groups. Other demographic characteristics not included in our questionnaire may have been of interest in determining factors that influence accuracy of men's self assessment of their balding pattern. These include factors such as marital status, occupation, and personality sub-types, and we would encourage any further research into the area to investigate the possible relationship between these aspects and the accuracy of men's self-assessment.

Any extrapolation of our data needs to take into account the differing methods of data collection between our study and other studies in which the man may obtain advice from partners or friends in the home environment, as well as have access to mirrors and photographs to assist in their assessment of current and past balding patterns. These factors do not negatively affect our results however, as the use of such help would ultimately increase accuracy of balding assessment from the already acceptable level shown in our results, rather than detract from it.

The study of the accuracy of previous balding was limited by small sample size. Participants had to be approached prior to the study to provide a photograph of them at age 35 years. This limited numbers of eligible participants, and also meant that the men may have viewed the photograph of themselves before completing the questionnaire, which may have increased their accuracy of retrospective assessment. Difficulties in assessing vertex balding from the photographs by the observer also became apparent, as often photographs did not provide an adequate view of the top and back section of a participant's head. It is unlikely that these limitations can be overcome, as it would be difficult to devise another method for the observer to retrospectively assess the participant's balding pattern at age 35.

Previous research has demonstrated an intra-observer rate of consistency in assessment of 98–99% using the Hamilton-Norwood scale[2,10]. Hamilton himself classified 200 balding patterns and then repeated the process three months later without reference to the original classifications. All but one of the classifications were identical. The scale was modified in 1975 by Norwood. The Hamilton scale has been found to correlate with local hair density[14]. A more simple classification of balding patterns was recently been described for use in hair restoration surgery[16] but this scale has not been used in the epidemiological context.

## Conclusions

From this study, we suggest that any further work requiring assessment of male balding patterns consider the use of trained observers as the gold standard of assessment. Where this is unattainable, we have shown that men's self evaluation is accurate enough to ensure reliability and validity of results. In addition, we believe this study demonstrates that if links are found between male balding patterns and health effects, that men can reliably determine their own balding pattern and assess their own risk.

## Competing interests

The author(s) declare that they have no competing interests.

## Authors contributions

RT and JM planned the study, collected the data, analysed the data under supervision, and wrote the study report for a student project.

JL assisted in planning the study and drafted the manuscript

LF assisted in planning the study, supervised data collection and analysis and edited the manuscript.

All authors read and approved the final manuscript

## Pre-publication history

The pre-publication history for this paper can be accessed here:


